# QTL Detection of Salt Tolerance at Soybean Seedling Stage Based on Genome-Wide Association Analysis and Linkage Analysis

**DOI:** 10.3390/plants13162283

**Published:** 2024-08-16

**Authors:** Maolin Sun, Tianxin Zhao, Shuang Liu, Jinfeng Han, Yuhe Wang, Xue Zhao, Yongguang Li, Weili Teng, Yuhang Zhan, Yingpeng Han

**Affiliations:** Key Laboratory of Soybean Biology in Chinese Ministry of Education (Key Laboratory of Soybean Biology and Breeding/Genetics of Chinese Agriculture Ministry), Northeast Agricultural University, Harbin 150030, China; smaolin2022@163.com (M.S.); ztxztx1111@163.com (T.Z.); 15047525832@163.com (S.L.); hjf272463@163.com (J.H.); wangyuhe@neau.edu.cn (Y.W.); xuezhao@neau.edu.cn (X.Z.); yongguangli@neau.edu.cn (Y.L.); twlneau@163.com (W.T.); zyhsoybean@163.com (Y.Z.)

**Keywords:** soybean, salt tolerance, GWAS, linkage analysis, molecular marker

## Abstract

The utilization of saline land is a global challenge, and cultivating salt-tolerant soybean varieties is beneficial for improving the efficiency of saline land utilization. Exploring the genetic basis of salt-tolerant soybean varieties and developing salt-tolerant molecular markers can effectively promote the process of soybean salt-tolerant breeding. In the study, the membership function method was used to evaluate seven traits related to salt tolerance and comprehensive salt tolerance at the soybean seedling stage; genome-wide association analysis (GWAS) was performed in a natural population containing 200 soybean materials; and linkage analysis was performed in 112 recombinant inbred lines (RIL) population to detect quantitative trait loci (QTLs) of salt tolerance. In the GWAS, 147 SNPs were mapped, explaining 5.28–17.16% of phenotypic variation. In the linkage analysis, 10 QTLs were identified, which could explain 6.9–16.16% of phenotypic variation. And it was found that there were two co-located regions between the natural population and the RIL population, containing seven candidate genes of salt tolerance in soybean. In addition, one colocalization interval was found to contain qZJS-15-1, rs47665107, and rs4793412, all of which could explain more than 10% of phenotypic variation rates, making it suitable for molecular marker development. The physical positions of rs47665107 and rs47934112 were included in qZJS-15-1. Therefore, a KASP marker was designed and developed using Chr. 15:47907445, which was closely linked to the qZJS-15-1. This marker could accurately and clearly cluster the materials of salt-tolerant genotypes in the heterozygous population tested. The QTLs and KASP markers found in the study provide a theoretical and technical basis for accelerating the salt-tolerant breeding of soybean.

## 1. Introduction

Soybean provides essential amino acids and abundant nutrients for humans and is also a potential biofuel raw material. It is widely used in industries such as food, feed, and processing. Twenty percent of the world’s arable land is affected by salinization, especially in arid and semi-arid regions, where soil salinization is becoming increasingly serious [1]. Soybean yield is affected by salt stress [2]. Exploring the genetic mechanism of salt tolerance in soybean, cultivating varieties of salt tolerance, and effectively utilizing saline-alkali soil are effective means to solve the problem of salt tolerance damage in soybean and improve soybean yield.

Salt stress has adverse effects on all stages of soybean growth and development [3]. Maintaining the survival rate and normal growth of soybean seedlings is a key factor in ensuring a high soybean yield. Therefore, a large number of reports have focused on the impact of salt stress on the soybean seedling stage. The study by Li et al. [4] showed that salt damage symptoms usually occur on soybean leaves, and within 10 min of being treated with NaCl their stomatal conductance decreased to less than 50% of the control. Ning et al. [5] found that the relative chlorophyll content, stomatal conductance, and transpiration rate of soybean leaves under salt stress would be significantly inhibited, and salt-sensitive soybeans would be more affected, with the degree of leaf wilt being much greater than in salt-tolerant soybean varieties. Begum et al. [6] found that the combined treatment of drought and salt reduced soybean plant height and root length, shoot and root total weights, relative water content, chlorophyll pigment, anthocyanin, and chlorophyll fluorescence content. In addition, it was found that under high salt stress, the Na^+^ content of soybean plants was significantly increased, but the K^+^, Ca^2+^, and Mg^2+^ contents were significantly reduced, and the activity of active oxygen scavenging enzymes was significantly reduced, leading to an increase in active oxygen species in a study by Amirjani et al. [7]. Deepening the research on the genetic mechanism of salt tolerance in soybean seedlings and applying it to soybean salt-tolerance breeding, cultivating salt-tolerant soybean varieties, is of great significance for increasing soybean yield.

Salt tolerance in soybean is a complex quantitative trait. QTL mapping is the main method for studying the genetic mechanism of quantitative traits. Linkage analyses are widely used in QTL mapping. Some reports on soybean salt-tolerance linkage analysis indicated the existence of a QTL affecting salt tolerance on chromosome 3. A study conducted by Lee et al. [8] detected a major QTL on soybean chromosome 3, mapped between Sat237 and Sat091, in the F2:5 population obtained by crossing salt-tolerant germplasm S-100 and salt-sensitive germplasm Tokyo. In another study, Hamwieh et al. [9] conducted linkage analysis on the F2 population obtained from the hybridization of the salt-tolerant germplasm JWS-156-1 and the salt-sensitive germplasm Jackson, confirming the study of Lee et al. [8]. Another similar study was carried out by Guan et al. [10] and the QTL on chromosome 3 was identified, and through further fine mapping, the gene *Glyma03g32900* was discovered. With the continuous deepening of research on soybean salt-tolerance linkage analysis, QTLs on other chromosomes have also been detected. For example, Fiskeby III and Williams 82 were used to construct an F_2_ population consisting of 132 strains for linkage analysis by Do et al. [11], and a novel salt-tolerant QTL was discovered on chromosome 13. Kefeng 1 and Nannong 1138-2 were used by Zhang et al. [12] to construct an RIL population for salt-tolerance linkage analysis, and a major QTL was detected on chromosome 8, and an important candidate gene, *GmCDF1* (*Glyma. 08g102000*), was discovered on the QTL. The F2:3 population constructed by soybean accessions, Beijing and NY36-87, was used for linkage analysis by GUO et al. [13] and mapped to a QTL on chromosome 18. In addition, the RIL population constructed from the salt-sensitive Korean cultivar, Qingjia 3, and the salt-tolerant landrace, IT162669, was utilized by Cho et al. [14] for salt-tolerance QTL detection, and 10 QTLs for six salt tolerance-related traits were detected on five chromosomes (Chr06, Chr09, Chr10, Chr13, and Chr17).

GWAS is also a common technique used for QTL mapping. A study was conducted by Zeng et al. [15], using leaf chloride ion concentration and chlorophyll content as indicators, to detect previously reported SNPs mapped on chromosomes 3 and chromosomes 2, as well as seven novel QTLs distributed on different chromosomes. In another study, Chlorophyll content, wilting grade of soybean leaves, and sodium ion content in leaves were identified as indicators by Do et al. [16], and GWAS were conducted through two independent populations, and the SNPs on chromosome 3 were detected in both populations, close to previously reported QTLs, while unidentified QTLs of previous studies were detected on chromosomes 1, 8, and 18. In addition, a study carried out by Wang et al. [17] detected SNPs related to soybean salt tolerance distributed on chromosomes 2, 5, 6, and 20, and designed KASP markers based on these SNPs. Recently, Chen et al. [18] used four salt tolerance-related traits of 286 soybean accessions for GWAS and found that almost all chromosomes contain SNPs related to salt tolerance.

The mapping of salt-tolerant QTLs in soybean was mainly focused on one of genome-wide association study or linkage analysis, and only a few studies used both methods to map salt-tolerant soybean. In fact, false positives cannot be completely avoided in GWAS or linkage analysis on QTL mapping, using GWAS combined with linkage analysis to conduct QTL mapping analysis, salt-tolerance QTLs of soybean can be more accurately detected. GWAS analysis combined with linkage analysis was used in this study to identify salt-tolerance QTLs in soybean, and the most important QTL detected was developed as a molecular marker and used in the screening of salt-tolerant soybean resources. The invention of salt-tolerant soybean varieties is an effective way to use saline alkali land reasonably and increase additional harvest. The QTL detected in the current study generated valuable knowledge on the genetic basis of salt tolerance, while the molecular markers developed will be of great significance for molecular-assisted breeding for salt tolerance in soybean.

## 2. Materials and Methods

### 2.1. Plant Material

A total of 200 landraces or elite cultivars were selected from the Chinese Soybean Gene Bank (CNSGB) for salt-tolerance evaluation and used for GWAS analysis. Based on the results of salt tolerance in natural populations, an RIL population was constructed using materials that showed differences in the seedling stage for the linkage analysis of soybean salt tolerance. The RIL population was a hybrid of Zhongdou 27 (salt-tolerant) and Jiunong 20 (salt-sensitive), consisting of 112 lines, which had been used for lodging linkage analysis in our previous studies [19]. This RIL population was referred to as the ZJ population in the paper. In addition, a heterozygous population was constructed by crossbreeding the salt-tolerant variety Zhongdou 27 to validate the QTL identified in the study and the developed molecular markers.

### 2.2. Evaluation of Traits Related to Salt Tolerance

The salt stress treatment experiment during the seedling stage was conducted in an artificial climate box, with constant light and temperature (constant temperature of 26 °C, light and dark cycle of 16 h/8 h). White light was used for plant growth with a light intensity of 300 μmol·m^−2^·s^−l^. The test material was planted in a culture tray with a 1:1 ratio of soil and vermiculite as the culture substrate. Five seeds were planted in each hole until the cotyledons of the plants were fully developed, and three seedlings with consistent growth were retained. In total, 10 mL of 150 mM NaCl was used for watering, once a day, for a total of three times. The salt-tolerance traits of plants were evaluated after 7 days, and we conducted 3 repeated experiments on each sample to obtain the average value.

Seven indicators, including chlorophyll content (CC), plant height (PH), root length (RL), above-ground fresh biomass (AGFB), below-ground fresh biomass (BGFB), above-ground dry biomass (AGDB), and below-ground dry biomass (BGDB), were used as salt-tolerance traits during the seedling stage to characterize the degree to which different test materials were affected by salt damage.

The membership function analysis method was used to evaluate the salt tolerance of different soybean resources during the seedling stage in the study. In the first stage, all indicators were converted into relative indicators, and the calculation formula was as follows: relative indicators = (treatment/control) × 100%. Afterwards, the membership function was used to evaluate the salt tolerance of different strains under different indicators. In addition, the average value of the membership functions of each trait (mean) was used to comprehensively evaluate the salt tolerance of the strain during the seedling stage. In the study, the larger the membership function value, the stronger the salt tolerance of the corresponding plant strain. The membership formula was as follows: *μ*(*Xj*) = (*Xj* − *Xmin*)/(*Xmax* − *Xmin*), and *j* = 1, 2, 3, …, *n*.

### 2.3. Genome-Wide Association Analysis

The CTAB method was used to extract genomic DNA from association analysis populations, and SLAF seq was used for SNP detection [20]. According to the threshold of MAF ≥ 5% and Missing ≤ 10%, polymorphic SNP filtering was performed, and ultimately 23,150 polymorphic SNP markers were obtained on 20 chromosomes of soybean.

Based on salt-tolerance traits at the seedling stage and 23,150 polymorphic SNPs, TASSEL 5.0 software [21] was used to conduct linkage disequilibrium analysis, principal component analysis (PCA), and kinship analysis on the whole genome association analysis population. Structure 2.3.4 software [22] was used to perform population structure analysis on the genome-wide association analysis population, estimate the optimal population size K, and set the K value range to 1–9, ΔK was used to determine the appropriate K value, and the specimen was assigned to the corresponding subgroup. In addition, the MLM (K + Q) model in TASSEL 5.0 software [21] was used for association analysis, with -log_10_(P) ≥ 3 [23] as the threshold for significant SNPs, and the results were plotted using the CM plot package in R studio.

In genome-wide association analysis, the range of 150 kbp upstream and downstream of the detected SNPs related to salt tolerance was used as the confidence interval for the corresponding QTL.

### 2.4. Linkage Analysis

The CTAB method [20] was used to extract DNA from RIL population pedigrees, and agarose gel electrophoresis was used to detect DNA. Qualified samples were used for subsequent construction of a Bin-map.

The construction of the Bin-map was completed by Biomarker Technologies (BMK) in Beijing. The sequencing process included library construction, library quality inspection, sequencing analysis, and SNP calling. The sliding window method [24] was used to merge SNPs within the minimum recombination interval into one marker block (bin), and based on the genotype typing results of the bins, a Bin-map genetic linkage map was constructed using QTL IciMapping v4.2 software [25]. The Complete Interval Mapping (ICIM) method in QTL IciMapping v4.2 software [25] was utilized to perform QTL mapping analysis on salt-tolerance traits in the RIL population, with a QTL threshold set to LOD > 2.5 [26].

In the study, the QTL was named as q + ZJ (abbreviations for the parental initials of RIL population) + S (seedling stage)—chromosome number—QTL number.

### 2.5. Prediction of Candidate Gene

The study compared the genetic overlap regions of the QTL found in the genome-wide association analysis and linkage analysis. SoyBase (http://www.soybase.org) was used to provide gene annotations for screening candidate genes in genetic overlap regions.

### 2.6. Development of Molecular Marker

Molecular marker assisted selection was performed on a heterozygous population containing Zhongdou 27 lineage using Chr. 15:47907445, a linked molecular marker of the salt-tolerant major QTL, qZJS-15-1, obtained in the study. The CTAB method [20] was used to extract genomic DNA, and the DNA quality was detected using a UV-2102C spectrophotometer. KASP markers were designed for Chr. 15:47907445, with primers as shown in Table S1. The primers were synthesized by the Ruibiotech company (Beijing, China).

The 7500 Software fluorescence quantitative PCR instrument was used for PCR reaction, with a reaction program of pre-denaturation at 95 °C for 10 min; Amplification Cycle 1: denaturation at 95 °C for 15 s, annealing at 61 °C–55 °C (−0.6 °C/cycles) for 60 s, a total of 10 cycles; Amplification Cycle 2: denaturation at 95 °C for 15 s, annealing at 55 °C for 60 s, a total of 32 cycles. The reaction system reference fluorescence quantitative reagent kit, FLU-ARMS for KASP 2x PCR Mix V4, was purchased from the Gude Biotechnology company in Guangzhou, China.

## 3. Results

### 3.1. Statistics and Analysis of Phenotype

**Figure 1:**
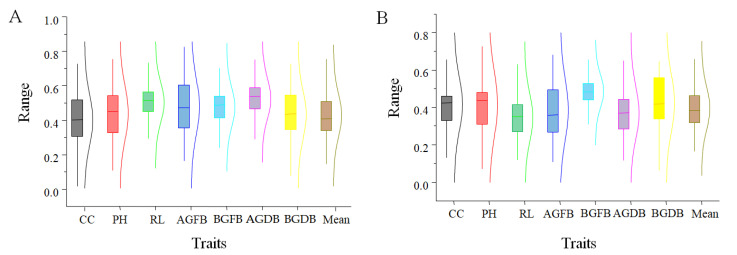
The membership function values of salt tolerance-related traits. (**A**) The box plot shows the range of membership function values for different traits of the natural population under salt treatment. (**B**) The box plot shows the range of membership function values for different traits of the RIL population under salt treatment.

In the study, a natural population composed of germplasm was subjected to salt stress treatment, and the salt tolerance of the population was analyzed through the membership functions of seven salt tolerance-related traits during the seedling stage and their average membership functions (Figure 1A and Table S2). Under salt stress conditions during the seedling stage, it was found that the membership function of PH had the largest range of variation, ranging from 0.04 to 0.79. The membership function of BGDB had the smallest range of variation, between 0.04 and 0.69. Statistics were conducted on the skewness and kurtosis of the membership functions and average membership functions of various traits under salt stress conditions during the seedling stage, and it was found that their absolute values were all less than one, showing a normal distribution. The RIL population (ZJ population) derived from the hybridization of Zhongdou 27 and Jiunong 20 was also subjected to salt stress in this study, and the membership functions and average membership functions (mean) of seven traits affected by salt stress during the seedling stage were statistically analyzed (Figure 1B and Table S2). Under salt stress during the seedling stage, it was found that the maximum range of variation in the membership function of PH among the selected traits was between 0.06 and 0.74, while the minimum range of variation in the membership function of BGFB was between 0.21 and 0.65. Statistics were conducted on the skewness and kurtosis of the membership functions of various traits and their average membership functions under salt stress conditions during the seedling stage, and it was found that their absolute values were all less than one, showing a normal distribution.

The correlation between the membership function values of seven salt-tolerance traits in natural populations during the seedling stage was statistically analyzed (Table 1). In the correlation analysis of PH, it was found that it was highly significantly correlated with RL, AGFB, BGFB, and AGDB (P < 0.01) and significantly correlated with BGDB (P < 0.05). In the correlation analysis of RL, it was found that it was highly significantly correlated with BGFB and BGDB and significantly correlated with AGDB. In the correlation analysis of AGFB, it showed a highly significant correlation with BGFB, AGDB, and BGDB, respectively. In the correlation analysis of BGFB, it showed a highly significant correlation with AGDB and BGDB, respectively. In the correlation analysis of AGDB, it was significantly correlated with BGDB. Although many of these traits had significant correlations, some of them had a lower range of phenotypic variation, which may result in imprecise phenotypic selection when used alone. In addition, it was found in the study that there was a correlation between comprehensive salt tolerance (mean) and various traits of salt tolerance, with a significant or extremely significant correlation coefficient.

### 3.2. Linkage Disequilibrium Analysis

**Figure 2:**
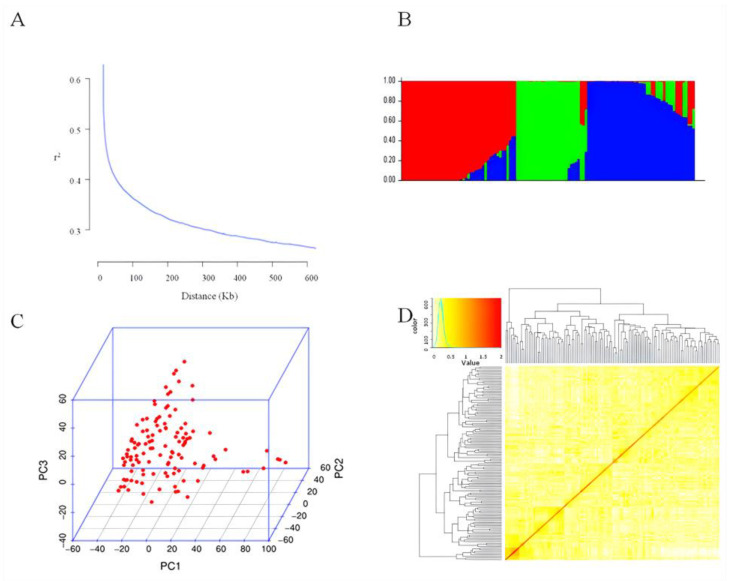
Linkage disequilibrium analysis, kinship, and population structure analysis. (**A**) Linkage disequilibrium analysis. (**B**) Population structure of varieties (K = 3). (**C**) Principal component analysis. (**D**) Kinship analysis.

In the study, 23,150 SNP markers were utilized with good polymorphism distributed on 20 chromosomes of soybean for GWAS analysis. By conducting linkage disequilibrium (LD) analysis on these SNPs, it was found that as the genetic distance increased, LD showed a rapid decay trend, with an average decay distance of approximately 200 kbp in the population (Figure 2A).

### 3.3. Kinship and Population Structure Analysis

In the study, group structure was analyzed, the maximum value of ΔK was used as a basis to determine suitable subgroups, and when ΔK was maximum, the number of subgroups was three, so it was considered that the natural population could be divided into three subgroups (Figure S1). In the Figure 2B, 200 experimental materials were divided into red, green, and blue groups, representing three subgroups, respectively. Principal component analysis (PCA) showed no significant stratification within the population, but the first three principal component analyses had a significant impact on population structure, which resulted in the population being divided into three subgroups in PCA analysis (Figure 2C). The analysis of genetic relationships showed that the genetic correlation between the 200 germplasm types was relatively small, which could effectively avoid genetic duplication (Figure 2D).

### 3.4. Genome-Wide Association Analysis of Salt Tolerance-Related QTLs

**Figure 3:**
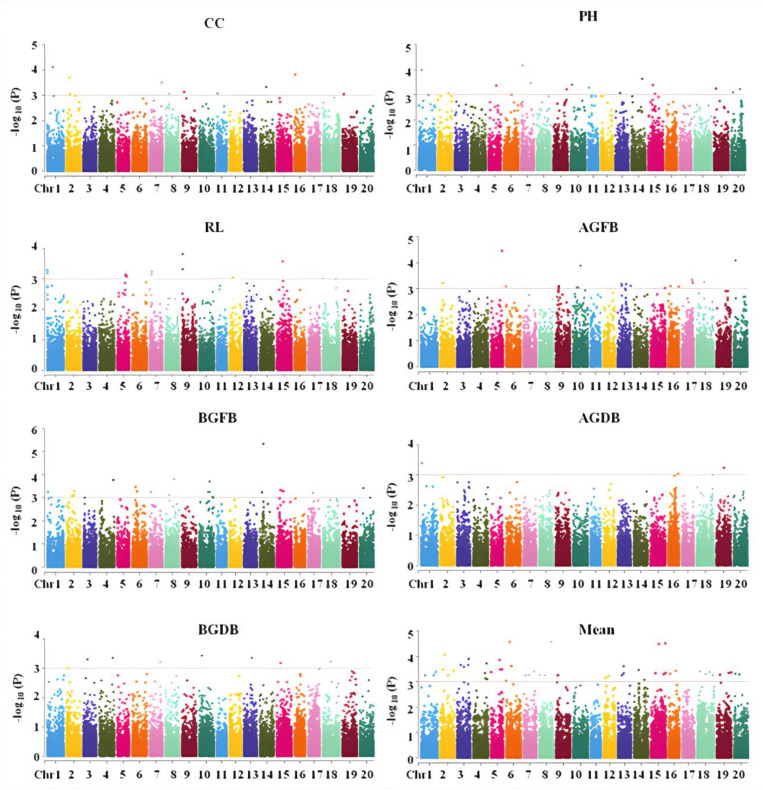
Distribution of SNPs related to salt tolerance on chromosomes of soybean.

This study utilized the strategy of whole genome association analysis to explore SNPs related to salt tolerance in the seedling stage. Using the membership functions of seven salt-tolerance traits and their average membership functions as indicators, a total of 147 SNPs were detected on 20 chromosomes of soybean, which could explain 5.28–17.16% of the phenotypic variation (Figure 3 and Table S3); 10 SNPs related to salt tolerance were detected in CC, which could explain phenotypic variations of 5.98–8.69%; 15 SNPs related to salt tolerance were detected in PH, which could explain phenotypic variations of 6.19–9.00%; 11 SNPs related to salt tolerance were detected in RL, which could explain phenotypic variations of 6.10–8.39%; 21 SNPs related to salt tolerance were detected in AGFB, which could explain phenotypic variations of 5.48–10.17%; 23 SNPs related to salt tolerance were detected in BGFB, which could explain phenotypic variations of 5.44–10.54%; 4 SNPs related to salt tolerance were detected in AGDB, which could explain phenotypic variations from 5.31% to 7.53%; 7 SNPs related to salt tolerance were detected in BGDB, which could explain phenotypic variations ranging from 5.28% to 7.03%; and in the mean membership function of each indicator, 55 SNPs related to salt tolerance were identified, which could explain phenotypic variations of 6.47–17.16%. Among these SNPs, rs19008986 on chromosome 1, rs13395916 on chromosome 8, rs47665107 on chromosome 15, and rs42306980 on chromosome 18 were mapped by at least two indicators. Among different indicators, a total of 10 SNPs had a phenotypic contribution rate exceeding 10%, which were the major QTLs affecting salt tolerance in soybean. And the phenotype contribution rate of rs47665107 mapped on chromosome 15 in the average membership function was the highest, at 17.16%.

### 3.5. Linkage Analysis of Salt Tolerance-Related QTLs

In this study, linkage analysis was used to detect QTLs related to salt tolerance in the ZJ population during the seedling stage. When LOD ≥ 2.5, QTLs related to salt tolerance were considered to exist (Figure S2 and Table 2). This study used the membership functions of seven salt-tolerant traits and their average membership functions as indicators to detect 10 QTLs distributed on six chromosomes, which could explain 6.9–16.16% of the phenotypic variation. The qZJS-2-1 detected on chromosome 2 could explain 13.95% of the phenotypic variation in CC. qZJS-13-1 detected on chromosome 13 could explain 11.78% of the phenotypic variation in PH. qZJS-16-1 detected on chromosome 16 could explain 12.31% of the phenotypic variation in RL. qZJS-1-1 detected on chromosome 1 could explain 12.23% of the phenotypic variation in AGFB. qZJS-15-1 detected on chromosome 15 could explain 14.00% of the phenotypic variation in BGFB. qZJS-10-1 detected on chromosome 10 could explain 16.16% of the phenotypic variation in AGDB. qZJS-13-2 detected on chromosome 13 could explain 11.43% of the phenotypic variation in BGDB. In the salt-tolerance localization using the mean membership function as an indicator, three QTLs were identified. Among them, qZJS-2-2 was mapped on chromosome 2, which could explain 6.9% of the phenotypic variation. qZJS-2-1 obtained in CC and qZJS-15-1 obtained in BGFB were also mapped in this indicator, which could explain 13.95% and 11.41% of the phenotypic variation.

### 3.6. Comparison of Salt Tolerance QTL Consistency Regions in Soybean and Screening of Candidate Genes

In this study, the range of 150 kbp upstream and downstream of the physical location of SNPs related to salt tolerance identified in natural populations was used as the confidence interval for the QTL corresponding to this SNP. And comparing the intervals of QTLs discovered in the natural population and those located in the RIL population, it was found that there were two colocalization regions between the natural population and the ZJ population under the same salt-tolerance index (Table 3). Eighty-five genes (Table S4) were co-located in two regions, and salt-tolerance candidate genes were screened using gene functional annotations provided on the SoyBase (http://www.soybase.org). Finally, seven genes were screened as candidate genes (Table 4). These genes were all known enzyme genes related to salt tolerance. Comparing qZJS-15-1 obtained through linkage analysis with rs47665107 and rs47934112 obtained through association analysis, it was found that three QTLs had consistent genomic segments (Table 3). And all of the three QTLs represented by qZJS-15-1, rs47665107, and rs47934112 could explain over 10% of the phenotypic variation rates. The intervals corresponding to rs47665107 and rs47934112 were included in qZJS-15-1; therefore, it was believed that qZJS-15-1 was suitable for molecular marker development.

### 3.7. Development and Validation of Salt-Tolerant Molecular Marker

The molecular marker Chr. 15:47907445, closely linked to the salt-tolerant main effector site qZJS-15-1, was used for the marker-assisted selection of heterozygous populations containing the Zhongdou 27 lineage. The study was based on Chr. 15:47907445 and designed AA and CC alleles as three KASP typing primers for population typing. Samples with the same genotype could be accurately and clearly clustered by this marker, which had a good effect on distinguishing different genotypes. Among them, the salt-tolerant germplasm was classified as CC or AA, and the negative control NT did not produce a signal (Figure S3). It could be seen from the KASP marker typing results of the population that the majority of the detected genotypes were AA, accounting for 85.5%, the genotype CC accounted for 4.5%, and heterozygous genotypes accounted for 10%. And the salt tolerance was analyzed for materials carrying the genotype CC, materials carrying genotype AA, and heterozygous materials. According to the t-test results, the difference in salt tolerance between the AA and CC genotypes reached a very significant level (P < 0.001, Figure S4). This marker had a good accuracy in assisting the selection of salt tolerance in soybean and could be a beneficial tool for the molecular-assisted breeding of salt tolerance in soybean.

## 4. Discussion

In order to accurately evaluate salt-tolerant soybean germplasm resources, different researchers have developed different salt-tolerance evaluation methods and salt-tolerance characterization indicators or salt-tolerance traits. Conducting salt-tolerance tests throughout the entire growth period in the field is considered to have the most important guiding significance for the actual production of soybeans, but it is easily affected by microclimate factors such as sunlight, temperature, precipitation, and wind, resulting in a low repeatability of the test results. Therefore, indoor testing methods and their characterization indicators are of great significance. There are many indicators of salt tolerance in soybeans, such as leaf chloride concentration and chlorophyll content, which were used by Zeng et al. [15] as characterization traits of soybean salt tolerance. The salt damage level and chlorophyll concentration were utilized by Shi et al. [26] as characterization traits of soybean salt tolerance. Some studies also used a few agronomic traits related to salt tolerance as evaluation indicators, but due to the limited number of indicators and their own characteristics, the results were relatively biased. In actual production, soybean seedlings, especially at the three-leaf and one-heart stage, are more sensitive to salt stress, and effective field management measures can be taken to alleviate it in various growth stages. Therefore, the study conducted indoor identification of salt tolerance for various traits affected by salt stress in soybean seedlings, and all traits exhibited rich variation and normal distribution during the seedling stage, laying a foundation for the exploration of genes with excellent salt tolerance.

GWAS and linkage analysis are the two most important research methods for studying the genetic mechanisms of quantitative traits [27]. GWAS is a technique based on linkage disequilibrium (LD) to detect genetic relationships between polymorphic markers and target traits in natural populations. In the study, the average LD decay distance of the population was approximately 200 kbp, which is at a relatively low level. A lower LD decay distance means that there are fewer candidate genes linked to the detected polymorphic markers that affect the target trait, thereby reducing the workload of candidate gene screening. Due to long-term natural selection and artificial domestication, soybean has formed a complex population structure, which can lead to false positives in GWAS. It is important to analyze the population structure before conducting GWAS. In the study, the population was divided into three subgroups, and the population structure was used as a covariate in the MLM model, which can improve the accuracy of GWAS. The construction of a molecular genetic map is the basis for conducting linkage analysis, and a high-density genetic map can efficiently detect salt-tolerance QTLs. In the study, a high-density Bin-map genetic linkage map based on resequencing technology containing 4112 bin markers was used to mine QTLs for salt tolerance at the seedling stage, laying the foundation for comprehensive scanning of the genetic background of salt-tolerant germplasm. In addition, two regions were found to be detected in both GWAS and linkage analysis under the same indicator in the study. GWAS can comprehensively analyze the genetic basis of salt tolerance, while linkage analysis reflects the genetic differences between parents in the genetic linkage population. GWAS or linkage analysis used alone cannot avoid the occurrence of false-positive QTLs [28]. By utilizing their respective advantages, comprehensive QTL detection can be carried out, effectively reducing false positives.

Many studies, both domestically and internationally, have identified a major QTL locus related to salt tolerance located on chromosome 3. A major QTL locus located near Sat237 and Sat091 on chromosome 3 was identified by Lee et al. [8]. The hybrid population constructed from wild soybeans was further confirmed by Hamwieh et al. [9], and two other genetic populations were also used by Hamwieh et al. [29] for salt-tolerance QTL mapping, both of which detected the major QTL on chromosome 3.The salt-tolerance gene corresponding to this QTL was determined by Guan et al. [10], and the QTL interval was narrowed down to 17.5 kb through fine mapping. And there was only one candidate gene in this interval, namely *Glyma03g32900*. In the study, GWAS analysis was used to locate some QTLs near the area, confirming the authenticity of this section. In addition, the study utilized whole genome association analysis to map rs29094555 on chromosome 13, which coincided with the salt-tolerant QTL identified by Zeng et al. [30]. And using linkage analysis, qZJS-2-1 on chromosome 2 was mapped close to the salt-tolerant SNP location identified by Do et al. [16].

Soybean salt-tolerance genes can be classified into ion transport genes, transcription factor genes, reactive oxygen species balance genes, and other genes. Ion transport genes included protein-coding genes involved in Na^+^ transport [10,31,32], protein-coding genes involved in Cl^-^ transport [4], and protein-coding genes involved in K^+^ transport [33]. Transcription factor genes include MYB, ERF, NAC, bZIP, and WRKY [34,35,36,37]. Reactive oxygen species balance genes include genes that increase the intracellular antioxidant content or enzyme activity [38,39]. Other genes include genes related to calmodulin and class B calmodulin [40,41], some kinase genes related to salt tolerance [42,43], aquaporin genes [44,45], small RNA [46], etc. The study selected regions where loci were co-located among different populations for predicting candidate genes and utilized SoyBase (http://www.soybase.org). Based on the location information of genes provided by Glyma 2.0, combined with gene function annotations on the website, a total of 7 candidate genes were predicted from 85 genes detected in two regions in GWAS and linkage analysis under the same indicators. These genes regulate the antioxidant content or enzyme activity in cells.

Molecular markers are a new and more ideal form of genetic markers developed after morphological, cellular, and biochemical markers [47]. Molecular markers have the advantages of being abundant, highly polymorphic, and not limited by seasons and environments, making them of significant application value in life science theory and research. At present, there are few reports on the development of salt-tolerant molecular markers in soybean. In this study, qZJS-15-1 obtained through linkage analysis had a consistency interval with rs47665107 and rs47934112 obtained through association analysis, and qZJS-15-1, rs47665107, and rs47934112, all three loci, could explain over 10% of the phenotypic variation rates. The positions of rs47665107 and rs47934112 were included in qZJS-15-1; therefore, qZJS-15-1 was selected for molecular marker development. The developed molecular markers were used for screening salt-tolerant materials, which could quickly screen materials with salt tolerance in specific populations. It not only provides support for soybean salt tolerance molecular-assisted breeding, but also proves the accuracy of the QTL identified in the study.

## Figures and Tables

**Table 1:** Correlation coefficients between traits.

	CC	PH	RL	AGFB	BGFB	AGDB	BGDB	Mean
CC	1							
PH	−0.09	1						
RL	−0.05	0.31 **	1					
AGFB	−0.07	0.26 **	0.0265	1				
BGFB	−0.03	0.31 **	0.32 **	0.38 **	1			
AGDB	0.013	0.23 **	0.19 *	0.49 **	0.37 **	1		
BGDB	−0.14	0.23 *	0.28 **	0.30 **	0.68 **	0.2 *	1	
Mean	0.21 *	0.32 **	0.23 *	0.35 **	0.23 *	0.21 *	0.35 **	1

Note: * and ** significant at 0.05 and 0.01 level.

**Table 2:** Statistics of salt-tolerance QTLs in the RIL population at the seedling stage.

Trait Name	Chromosome	Genetic Position (cM)	Range (cM) ^A^	Interval (bp) ^B^	LOD	PVE (%) ^C^	QTL Name
CC	2	22	20.5–23.5	43637691–43932528	2.69	13.95	qZJS-2-1
PH	13	45	44.5–46.5	33207771–33517956	2.52	11.78	qZJS-13-1
RL	16	28	26.5–28.5	5342596–5565208	2.58	12.31	qZJS-16-1
AGFB	1	43	42.5–43.5	43519723–43535518	2.52	12.23	qZJS-1-1
BGFB	15	129	128.5–129.5	47731881–48413184	2.62	14	qZJS-15-1
AGDB	10	37	36.5–37.5	37207079–37242844	3.04	16.16	qZJS-10-1
BGDB	13	98	95.5–99.5	15309561–15581977	2.9	11.43	qZJS-13-2
Mean	2	22	20.5–23.5	43637691–43932528	6.69	13.95	qZJS-2-1
	2	74	73.5–74.5	10143604–10143635	3.68	6.9	qZJS-2-2
	15	94	92.5–97.5	47731881–48413184	5.48	11.41	qZJS-15-1

^A^ Support interval of the genetic position. ^B^ Interval of physical position of SNP makers. ^C^ Phenotypic variance explained by individual QTLs.

**Table 3:** Colocalization of QTLs among populations.

Stage	Name	Chromosome	Trait	Population	PVE (%) ^A^
Seedling stage	rs43642631, qZJS-2-1	2	CC	Natural population, RIL population	5.98, 13.95
	rs47665107, rs47934112, qZJS-15-1	15	Mean	Natural population, RIL population	17.16, 12.08, 11.41

^A^ Phenotypic variance explained by individual QTLs.

**Table 4:** Candidate genes.

Stage	Gene	Chromosome	Gene Function
Seedling stage	*Glyma.02g249300*	2	Glycerol-3-phosphate acyltransferase 5
	*Glyma.02g249600*	2	Rubisco activase
	*Glyma.02g250200*	2	Protein phosphatase 2CA
	*Glyma.15G250100*	15	nine-cis-epoxycarotenoid dioxygenase 3
	*Glyma.15G250200*	15	receptor-like protein kinase 1
	*Glyma.15G251300*	15	Nicotianamine synthase 1
	*Glyma.15G252600*	15	Leucine-rich receptor-like protein kinase family protein

## Data Availability

Data are contained within the article and the Supplementary Materials.

## Supplementary Materials

The following supporting information can be downloaded at https://www.mdpi.com/article/10.3390/plants13162283/s1.
