# Supplementary material for: QTL Detection of Salt Tolerance at Soybean Seedling Stage Based on Genome-Wide Association Analysis and Linkage Analysis
Source: Plants (Basel). 2024 Aug 16;13(16):2283. doi: 10.3390/plants13162283 (PMC11360379; doi:10.3390/plants13162283)
Supplement: Supplementary file 1 [file plants-13-02283-s001.zip › plants-3121124-supplementary.pdf]

plants

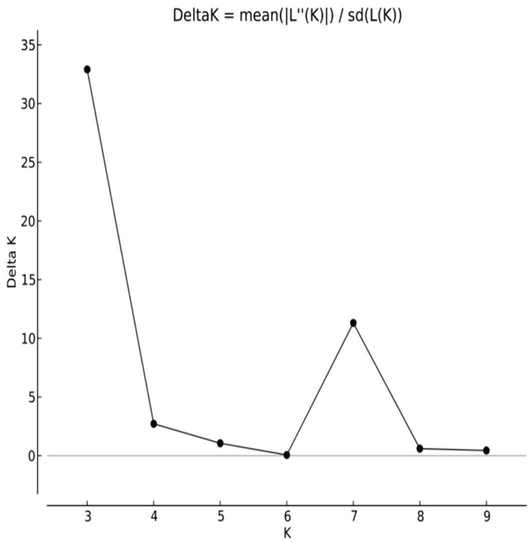

Figure S1. Change curve of △K value with K value.

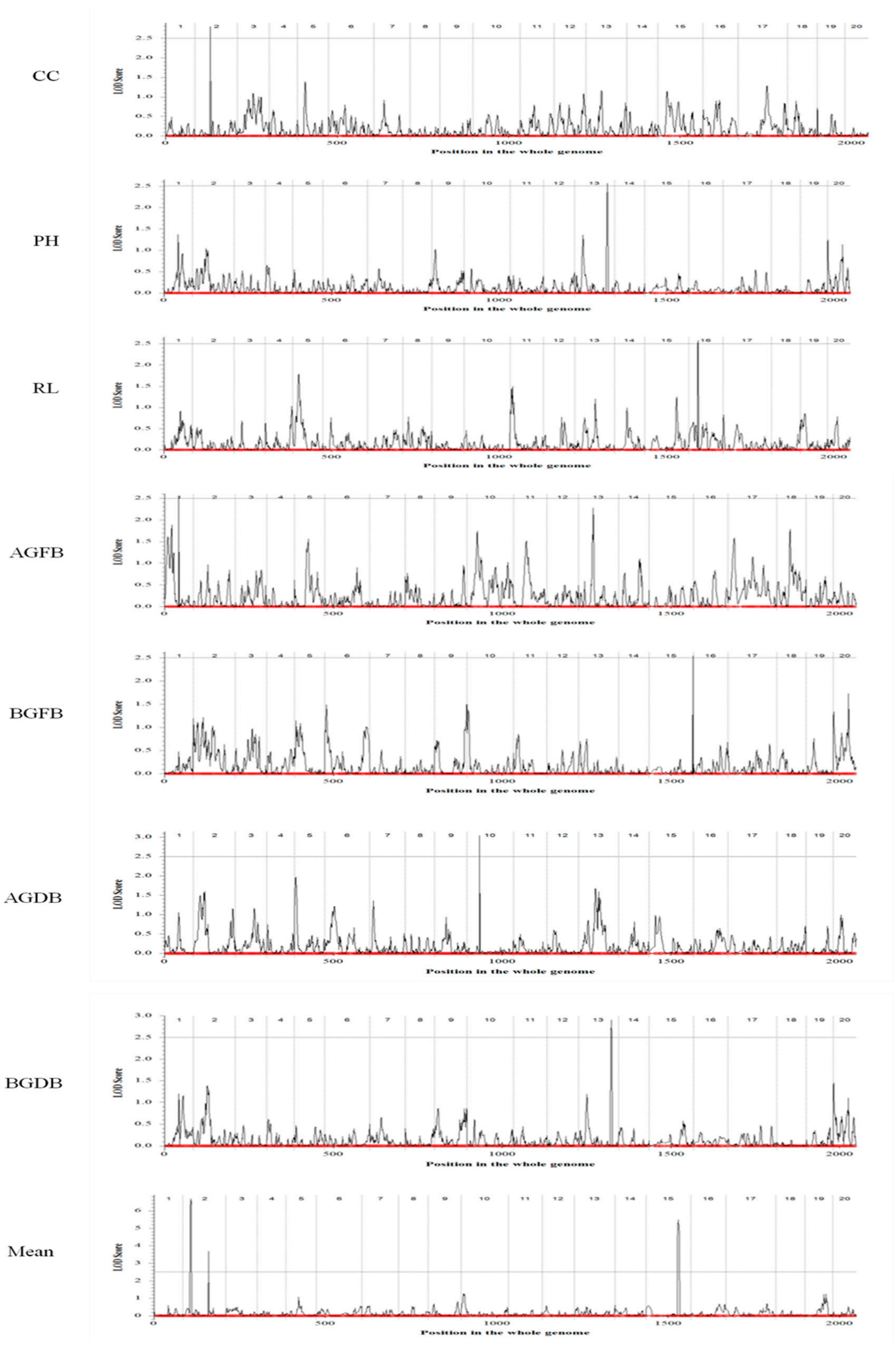

Figure S2. Chromosome distribution of salt tolerant QTL in RIL population at seedling stage.

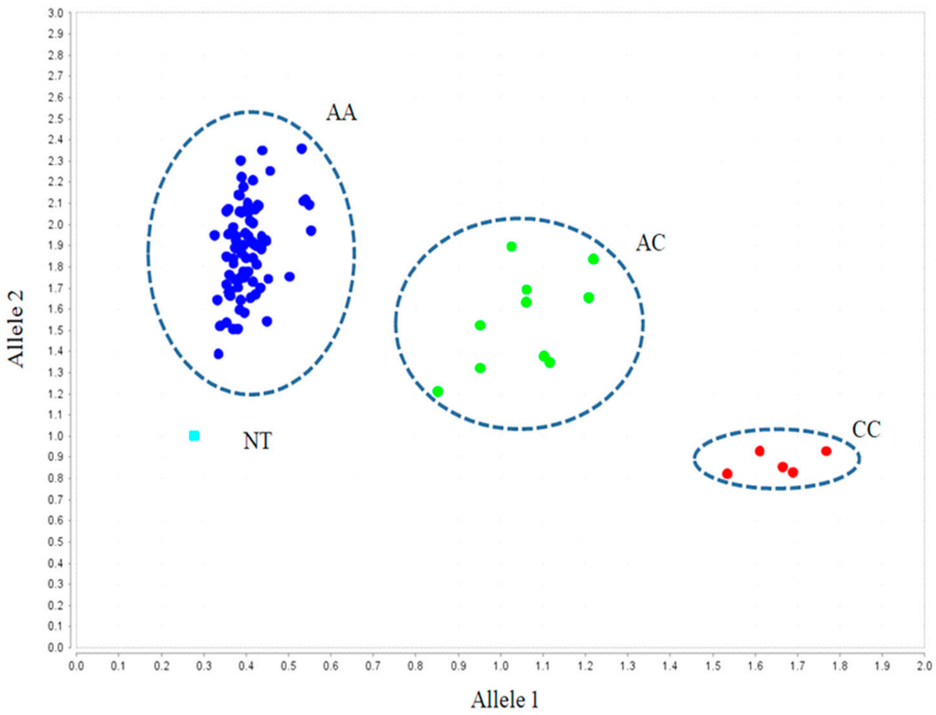

Figure S3. The effect of KASP marker typing.

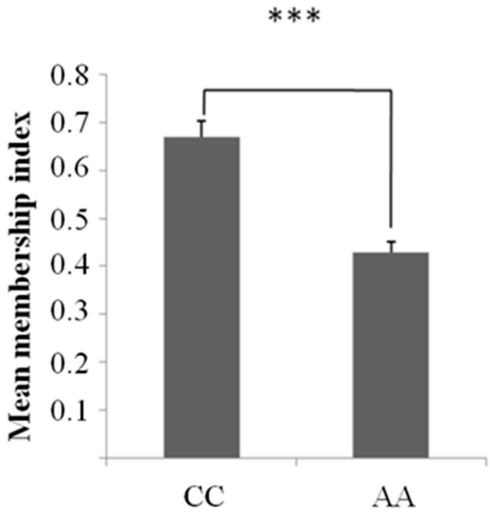

Figure S4. Genotyping of KASP markers**.** Effect analysis of KASP marker in assisting selection of salt tolerant germplasm; * * *: Significant at 0.001 level.

Table S1. KASP marker primer sequence.

Primername	Fluorophore	Sequence
qTSS-15-1-Allele1	FAM	**GAAGGTGACCAAGTTCATGCT**GTAAGTTCTATGCAAGTGA**C**
qTSS-15-1-Allele2	HEX	**GAAGGTCGGAGTCAACGGATT**GTAAGTTCTATGCAAGTGA**A**
qTSS-15-1-Common		GGAGCCTGAGAGAATGGACC

Table S2. Phenotypic analysis of natural population and RIL population under salt treatment.

Category	Traits	Mean	SD[A]	Range	Skewness	Kurtosis	CV(%)[B]
Naturalpopulation	CC	0.41	0.17	0.03-0.75	0.38	0.64	0.41
	PH	0.44	0.11	0.04-0.79	0.14	0.32	0.25
	RL	0.51	0.19	0.05-0.78	0.15	0.41	0.37
	AGFB	0.42	0.16	0.11-0.81	-0.25	0.23	0.38
	BGFB	0.47	0.22	0.08-0.76	0.45	0.46	0.47
	AGDB	0.44	0.19	0.11-0.79	0.23	0.44	0.43
	BGDB	0.41	0.15	0.04-0.69	0.38	0.57	0.37
	Mean	0.38	0.14	0.07-0.74	-0.14	0.64	0.37
RIL population	CC	0.42	0.14	0.11-0.71	0.52	0.74	0.33
	PH	0.43	0.12	0.06-0.74	-0.15	0.11	0.28
	RL	0.37	0.16	0.13-0.68	0.35	0.21	0.43
	AGFB	0.38	0.14	0.13-0.68	-0.55	0.53	0.37
	BGFB	0.49	0.19	0.23-0.75	0.25	0.66	0.39
	AGDB	0.39	0.16	0.11-0.64	0.03	0.24	0.41
	BGDB	0.42	0.22	0.08-0.65	0.45	0.37	0.52
	Mean	0.4	0.16	0.15-0.68	-0.44	0.24	0.4

[A]Standard deviation. [B]Coefficient of variation.

Table S3. Statistics of SNP related to salt tolerance in soybean seedling stage.

Trait name	Chromosome	SNP name	Position (bp)	P	R^2(%)
CC	1	rs19008986	19008986	7.71E-05	7.26
	16	rs2645399	2645399	1.53E-04	7.75
	2	rs9562214	9562214	1.98E-04	7.49
	7	rs41516223	41516223	3.15E-04	8.69
	14	rs22162172	22162172	4.73E-04	8.25
	9	rs7820697	7820697	7.36E-04	7.77
	11	rs2697043	2697043	8.47E-04	6.05
	8	rs13395918	13395918	8.79E-04	6.01
	19	rs4988221	4988221	8.99E-04	7.56
	2	rs43642631	43642631	9.04E-04	5.98
PH	7	rs5100826	5100826	6.89E-05	7.79
	1	rs9867480	9867480	1.04E-04	9
	14	rs37374928	37374928	2.30E-04	8.21
	7	rs33076691	33076691	3.38E-04	7.83
	10	rs4284269	4284269	3.98E-04	6.19
	15	rs15838410	15838410	4.11E-04	7.64
	5	rs26645993	26645993	4.34E-04	6.11
	11	rs2241188	2241188	5.17E-04	7.41
	19	rs6981881	6981881	5.66E-04	7.32
	20	rs29979450	29979450	5.95E-04	7.28

	9	rs46108403	46108403	6.03E-04	7.26
	20	rs6221329	6221329	7.96E-04	6.99
	13	rs16412503	16412503	8.50E-04	6.93
	2	rs35674944	35674944	8.62E-04	6.92
	15	rs23130653	23130653	9.12E-04	6.86
RL	9	rs2511987	2511987	1.53E-04	7.97
	15	rs20556803	20556803	2.66E-04	7.39
	9	rs2751661	2751661	4.81E-04	6.78
	1	rs477407	477407	5.13E-04	8.39
	7	rs7602728	7602728	5.75E-04	6.6
	1	rs645875	645875	6.39E-04	6.49
	7	rs7382554	7382554	7.07E-04	8.03
	5	rs29342005	29342005	7.35E-04	6.35
	5	rs32049087	32049087	8.24E-04	7.86
	12	rs11171111	11171111	8.84E-04	6.17
	18	rs803907	803907	9.49E-04	6.1
AGFB	5	rs38068226	38068226	3.54E-05	10.17
	20	rs6864965	6864965	8.21E-05	9.31
	10	rs26167101	26167101	1.30E-04	8.84
	17	rs34451631	34451631	4.52E-04	7.6
	18	rs25936597	25936597	5.52E-04	7.4
	17	rs37446027	37446027	5.57E-04	5.93
	17	rs36769430	36769430	6.10E-04	5.84
	2	rs9380907	9380907	6.15E-04	7.29
	13	rs13155244	13155244	6.60E-04	7.22
	13	rs29094555	29094555	6.68E-04	5.76
	13	rs14078983	14078983	7.74E-04	7.07
	13	rs44971576	44971576	7.80E-04	7.06
	9	rs11123116	11123116	7.96E-04	7.04
	16	rs8140663	8140663	7.97E-04	5.61
	6	rs789641	789641	8.22E-04	7.01
	16	rs36300581	36300581	8.40E-04	6.99
	10	rs13892467	13892467	8.74E-04	6.95
	10	rs18059175	18059175	9.19E-04	5.48
	15	rs47665107	47665107	3.07E-05	13.16
	9	rs11031949	11031949	9.50E-04	6.87
	13	rs26132490	26132490	9.92E-04	6.83
BGFB	14	rs12398785	12398785	4.57E-06	10.54
	8	rs30214972	30214972	1.52E-04	7.15
	4	rs48368354	48368354	1.70E-04	8.62
	10	rs36018964	36018964	1.95E-04	6.92
	6	rs12025740	12025740	3.40E-04	7.93
	20	rs12497371	12497371	3.84E-04	7.81

	15	rs13284857	13284857	4.70E-04	7.6
	2	rs26594655	26594655	5.12E-04	7.52
	15	rs22703309	22703309	5.20E-04	7.5
	6	rs16973678	16973678	5.29E-04	6.01
	1	rs2194375	2194375	5.57E-04	5.96
	7	rs4283227	4283227	5.57E-04	5.96
	10	rs31655849	31655849	5.57E-04	5.96
	14	rs7620528	7620528	5.81E-04	7.39
	17	rs16440855	16440855	6.06E-04	7.35
	8	rs13395916	13395916	7.61E-04	5.68
	2	rs24650266	24650266	7.87E-04	7.09
	2	rs19298170	19298170	8.14E-04	7.06
	10	rs46085553	46085553	9.28E-04	6.93
	1	rs19008986	19008986	9.67E-04	6.89
	10	rs49178317	49178317	9.84E-04	6.88
	20	rs35328708	35328708	9.95E-04	6.86
	3	rs4504431	4504431	9.97E-04	5.44
AGDB	1	rs3079635	3079635	4.08E-04	7.53
	19	rs26870613	26870613	5.79E-04	7.2
	16	rs33948525	33948525	9.03E-04	5.38
	18	rs54593190	54593190	9.83E-04	5.31
BGDB	10	rs9380593	9380593	3.67E-04	6.12
	4	rs46509141	46509141	4.35E-04	7.42
	13	rs27567869	27567869	4.37E-04	7.42
	3	rs14445672	14445672	4.94E-04	5.86
	18	rs27066313	27066313	5.90E-04	7.13
	7	rs36787161	36787161	6.15E-04	5.67
	15	rs13370032	13370032	6.54E-04	7.03
	2	rs6771042	6771042	9.67E-04	5.28
Mean	8	rs41540967	41540967	2.64E-05	12.35
	6	rs12370098	12370098	2.71E-05	12.32
	15	rs47665107	47665107	3.07E-05	17.16
	15	rs47934112	47934112	3.29E-05	12.08
	2	rs15264832	15264832	8.64E-05	10.88
	3	rs39895210	39895210	1.26E-04	10.42
	5	rs39657274	39657274	1.39E-04	10.3
	4	rs46015605	46015605	1.90E-04	9.93
	3	rs38224459	38224459	2.20E-04	9.75
	3	rs12488215	12488215	2.21E-04	9.74
	6	rs18591894	18591894	2.42E-04	9.63
	13	rs19394331	19394331	2.46E-04	9.61
	3	rs23391671	23391671	2.61E-04	9.54
	2	rs12597432	12597432	3.25E-04	9.28

5	rs38377329	38377329	3.25E-04	9.28
5	rs31443599	31443599	3.32E-04	7.46
14	rs16115212	16115212	3.45E-04	9.21
2	rs45855719	45855719	3.60E-04	9.16
16	rs24669050	24669050	3.72E-04	9.12
18	rs9163982	9163982	3.97E-04	9.05
7	rs36678209	36678209	3.99E-04	9.04
1	rs50334843	50334843	4.15E-04	8.99
18	rs42306980	42306980	4.15E-04	8.99
5	rs287293	287293	4.16E-04	8.99
19	rs50025718	50025718	4.30E-04	8.95
19	rs41279002	41279002	4.52E-04	8.89
15	rs50765206	50765206	4.53E-04	8.89
4	rs40908100	40908100	4.60E-04	7.1
13	rs19394310	19394310	4.64E-04	8.86
15	rs15417769	15417769	4.67E-04	8.85
15	rs15983665	15983665	4.71E-04	8.84
16	rs5540602	5540602	4.85E-04	7.05
20	rs4397102	4397102	4.85E-04	7.05
18	rs29780369	29780369	4.95E-04	8.78
15	rs43704820	43704820	5.11E-04	8.75
20	rs18173239	18173239	5.20E-04	8.73
19	rs11526203	11526203	5.30E-04	8.71
8	rs933629	933629	5.45E-04	8.67
7	rs19473462	19473462	5.46E-04	8.67
8	rs13395916	13395916	5.56E-04	8.65
9	rs5630582	5630582	5.56E-04	8.65
13	rs13180450	13180450	5.56E-04	8.65
2	rs26833731	26833731	5.57E-04	8.65
1	rs43109643	43109643	5.58E-04	8.64
18	rs42306980	42306980	5.58E-04	8.64
7	rs6987792	6987792	5.59E-04	6.9
1	rs19008986	19008986	5.60E-04	8.64
9	rs8067942	8067942	5.61E-04	8.64
12	rs16544894	16544894	6.00E-04	8.56
12	rs6479122	6479122	6.94E-04	8.39
4	rs42208812	42208812	7.45E-04	8.31
4	rs47986756	47986756	7.84E-04	6.54
4	rs47849623	47849623	8.04E-04	8.22
14	rs35784850	35784850	8.37E-04	6.47
6	rs84943	84943	9.19E-04	8.06

Table S4. Functions of genes included in the consistency interval.

Gene	Chromosome	Function
Glyma.02g248900	2	ROTUNDIFOLIA like 8
Glyma.02g249000	2	Eukaryotic aspartyl protease family protein
Glyma.02g249100	2	RING-H2 finger C2A
Glyma.02g249200	2	Unknown
Glyma.02g249300	2	Glycerol-3-phosphate acyltransferase 5
Glyma.02g249400	2	RAD-like 6
Glyma.02g249500	2	Pathogenesis-related thaumatin superfamily protein
Glyma.02g249600	2	Rubisco activase
Glyma.02g249700	2	LOB domain-containing protein 33
Glyma.02g249800	2	AT-hook motif nuclear-localized protein 20
Glyma.02g249900	2	Unknown
Glyma.02g250000	2	Sec7 domain-containing protein
Glyma.02g250100	2	Zinc-finger protein 10
Glyma.02g250200	2	Protein phosphatase 2CA
Glyma.02g250300	2	Unknown
Glyma.02g250400	2	S-adenosyl-L-methionine-dependent methyltransferases superfamily protein
Glyma.02g250500	2	Unknown
Glyma.02g250600	2	Pentatricopeptide repeat (PPR-like) superfamily protein
Glyma.02g250700	2	Regulator of chromosome condensation (RCC1) family protein
Glyma.02g250800	2	Glucose-1-phosphate adenylyltransferase family protein
Glyma.02g250900	2	Histone acetyltransferase of the GNAT family 2
Glyma.02g251000	2	Mitochondrial glycoprotein family protein
Glyma.02g251100	2	Delta 1-pyrroline-5-carboxylate synthase 2
Glyma.02g251200	2	Integral membrane Yip1 family protein
Glyma.02g251300	2	Exostosin family protein
Glyma.02g251400	2	DHFS-FPGS homolog B
Glyma.02g251500	2	Transducin/WD40 repeat-like superfamily protein
Glyma.02g251600	2	Type one serine/threonine protein phosphatase 4
Glyma.02g251700	2	Nucleotide binding;protein binding
Glyma.02g251800	2	DA1-related protein 2
Glyma.02g251900	2	Mitochondrial substrate carrier family protein
Glyma.02g252000	2	RAB GTPase homolog A2B
Glyma.02g252100	2	Unknown
Glyma.02g252200	2	Subtilase family protein
Glyma.15g250100	15	Nine-cis-epoxycarotenoid dioxygenase 3
Glyma.15g250200	15	receptor-like protein kinase 1
Glyma.15g250300	15	Unknown
Glyma.15g250400	15	Spc97 / Spc98 family of spindle pole body (SBP) component
Glyma.15g250500	15	TCP-1/cpn60 chaperonin family protein
Glyma.15g250600	15	Photosystem II reaction center PSB29 protein
Glyma.15g250700	15	Exocyst subunit exo70 family protein C2
Glyma.15g250800	15	Aldolase-type TIM barrel family protein

Glyma.15g250900	15	S-adenosyl-L-methionine-dependent methyltransferases superfamily protein
Glyma.15g251000	15	Nucleotide/sugar transporter family protein
Glyma.15g251100	15	Ubiquitin-specific protease 25
Glyma.15g251200	15	Unknown
Glyma.15g251300	15	Nicotianamine synthase 1
Glyma.15g251400	15	Unknown
Glyma.15g251500	15	Glutathione S-transferase TAU 19
Glyma.15g251600	15	Glutathione S-transferase TAU 19
Glyma.15g251700	15	Glutathione S-transferase TAU 19
Glyma.15g251800	15	Glutathione S-transferase TAU 19
Glyma.15g251900	15	Glutathione S-transferase TAU 19
Glyma.15g252000	15	Glutathione S-transferase TAU 19
Glyma.15g252100	15	Glutathione S-transferase TAU 19
Glyma.15g252200	15	Glutathione S-transferase TAU 19
Glyma.15g252300	15	Glutathione S-transferase TAU 19
Glyma.15g252400	15	Unknown
Glyma.15g252500	15	Glutathione S-transferase TAU 20
Glyma.15g252600	15	Leucine-rich receptor-like protein kinase family protein
Glyma.15g252700	15	WAG 1
Glyma.15g252800	15	Di-glucose binding protein with Kinesin motor domain
Glyma.15g252900	15	Unknown
Glyma.15g253000	15	Translation initiation factor 2, small GTP-binding protein
Glyma.15g253100	15	IAP-like protein 1
Glyma.15g253200	15	cAMP-regulated phosphoprotein 19-related protein
Glyma.15g253300	15	Chaperone DnaJ-domain superfamily protein
Glyma.15g253400	15	Blue-copper-binding protein
Glyma.15g253500	15	Protein kinase superfamily protein
Glyma.15g253600	15	Vacuolar ATP synthase catalytic subunit-related / V-ATPase-related / vacuolar proton pump-related
Glyma.15g253700	15	Photosystem II subunit R
Glyma.15g253800	15	U5 small nuclear ribonucleoprotein helicase, putative
Glyma.15g253900	15	DNAse I-like superfamily protein
Glyma.15g254000	15	NAC domain containing protein 1
Glyma.15g254100	15	Ribosomal protein L1p/L10e family
Glyma.15g254200	15	Protein of unknown function (DUF1218)
Glyma.15g254300	15	Disease resistance family protein / LRR family protein
Glyma.15g254400	15	Unknown
Glyma.15g254500	15	SNARE-associated protein-related
Glyma.15g254600	15	Small nuclear ribonucleoprotein family protein
Glyma.15g254700	15	Pathogenesis-related thaumatin superfamily protein
Glyma.15g254800	15	Pentatricopeptide repeat (PPR) superfamily protein
Glyma.15g254900	15	Pentatricopeptide repeat (PPR) superfamily protein
